# Persistent Acral Pityriasis Rosea in a Pediatric Patient: A Rare Clinical Variant

**DOI:** 10.7759/cureus.105958

**Published:** 2026-03-27

**Authors:** Lana T Alsemairi, Asail S Alghamdi, Muath Alghamdi, Abdullah Alghamdi, Saleh Alghamdi, Mohammed Alzahrani

**Affiliations:** 1 Dermatology, Uhud General Hospital, Madinah, SAU; 2 Dermatology, King Fahad Hospital, Albaha, SAU; 3 Dermatology, Albaha Health Cluster, Albaha, SAU

**Keywords:** acral involvement, papulosquamous disorders, pediatric dermatology, persistent pityriasis rosea, pityriasis rosea

## Abstract

Pityriasis rosea (PR) is a self-limited papulosquamous skin condition most commonly observed in children and young adults. Atypical variants of PR have been increasingly recognized, particularly those with unusual morphology, distribution, or disease course. Acral involvement is rare and may pose diagnostic challenges, especially in pediatric patients.

We report the case of a 15-year-old previously healthy Saudi male patient who presented with a persistent erythematous and scaly eruption lasting for more than three months. The eruption involved the trunk and extremities and showed prominent acral involvement affecting both the palms and soles. The patient denied pruritus, pain, recent infections, medication use, or similar previous episodes. Physical examination revealed multiple erythematous scaly plaques and patches without mucosal, nail, or lymph node involvement. Histopathological examination of a punch biopsy demonstrated findings consistent with PR in the appropriate clinical context. The patient was managed with narrowband ultraviolet B phototherapy, topical betamethasone combined with calcipotriol, urea 10% cream, and vitamin D supplementation. This case highlights a rare presentation of persistent acral PR in a pediatric patient, emphasizing the importance of recognizing atypical and prolonged presentations to avoid misdiagnosis.

## Introduction

Pityriasis rosea (PR) is an acute, self-limiting papulosquamous dermatosis that predominantly affects children and young adults [1]. It presents as scaly papules and plaques aligned along Langer’s lines (cleavage lines) on the trunk and extremities, typically forming a V-shaped pattern on the upper chest and a Christmas tree pattern on the back. The eruption is usually preceded by a herald patch [2,3]. A collarette scale, characterized by peripheral attachment with central lifting, is a classic feature of PR [4]. The exact etiology of PR remains uncertain. Clustering of cases, seasonal variation, prodromal symptoms, and a rash pattern resembling a viral exanthem (an initial herald patch followed by secondary eruptions), along with a low relapse rate, suggest a possible viral origin. In addition, immune-mediated mechanisms involving cytokines and hypersensitivity reactions to environmental triggers have also been proposed [5]. PR typically resolves spontaneously within six to eight weeks; however, in some cases, it may persist for up to three months [6]. Although PR most commonly presents in its classic form, atypical variants have been increasingly recognized. These variants are classified according to the presence or absence of a herald patch, lesion distribution, disease course, and morphology. They may be grouped by morphology (e.g., purpuric, urticarial, vesicular, follicular, hypopigmented, and gigantea), distribution (e.g., inverse, acral, unilateral, and Blaschkoid (i.e., following the lines of Blaschko)), and clinical course (e.g., relapsing, recurrent, and persistent). Recognition of these atypical presentations is important to prevent misdiagnosis and ensure appropriate management [7]. Here, we report a rare case of persistent acral PR in a pediatric patient.

## Case presentation

A 15-year-old previously healthy Saudi male patient presented with a three-month history of erythematous and scaly skin lesions involving the entire body, including the palms and soles. The lesions initially appeared on the trunk and gradually progressed to involve the extremities, including the palms and soles, with continued appearance of new lesions over time. He denied any associated symptoms such as itching or pain and has no history of recent infections, new medications, or similar prior episodes. Furthermore, the patient had no known personal or family history of dermatological or autoimmune diseases. He had not been previously diagnosed or treated for this condition prior to presentation.

**Figure 1:**
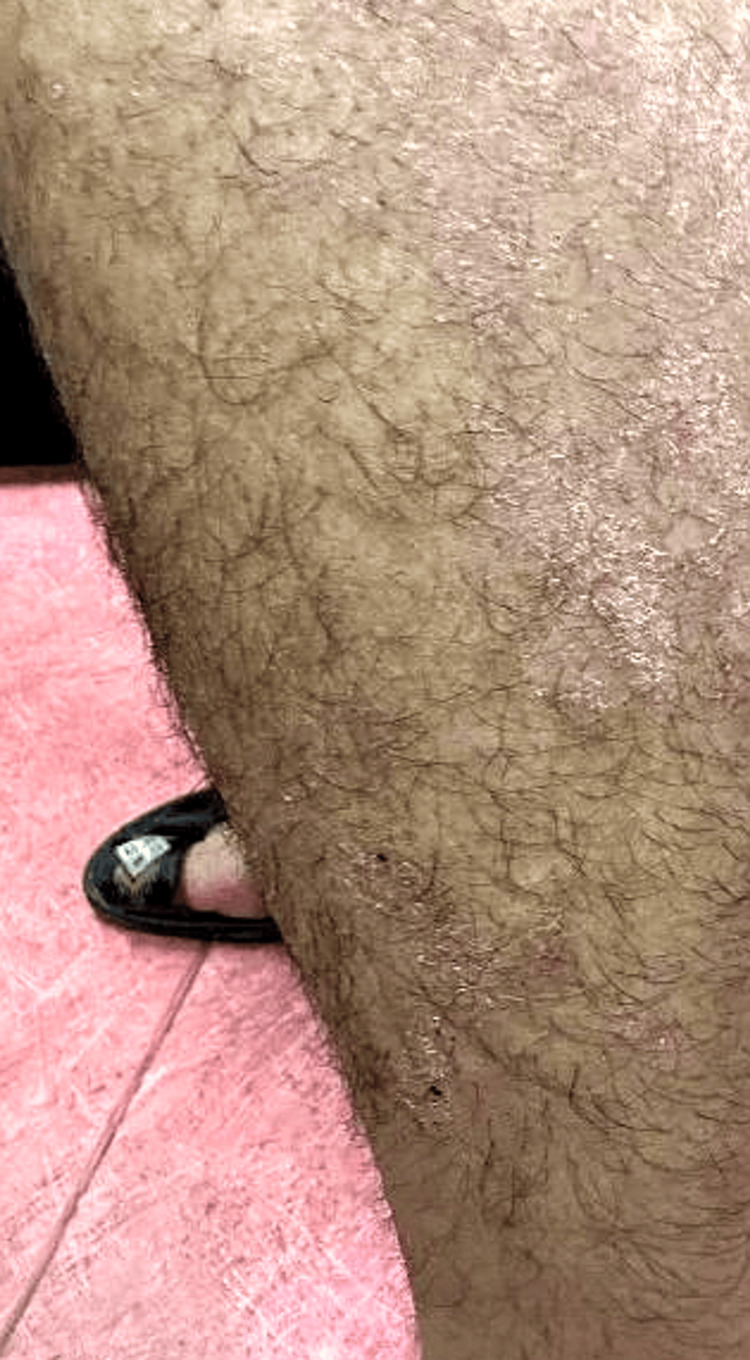
Multiple ill-defined erythematous scaly patches over the lateral aspect of one leg.

**Figure 2:**
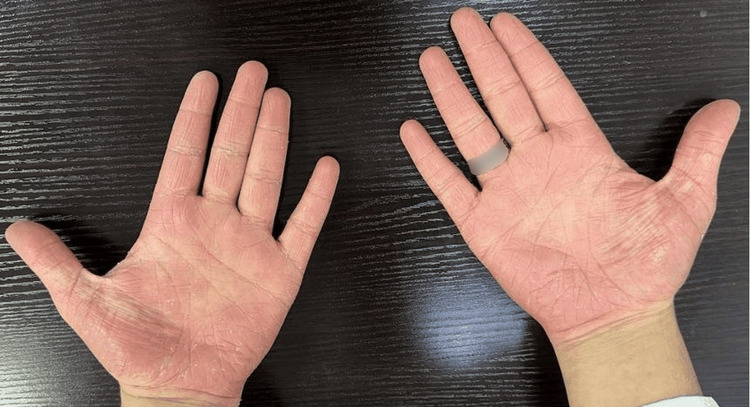
Ill-defined erythematous patches involving both palmar surfaces with collarette scaling.

**Figure 3:**
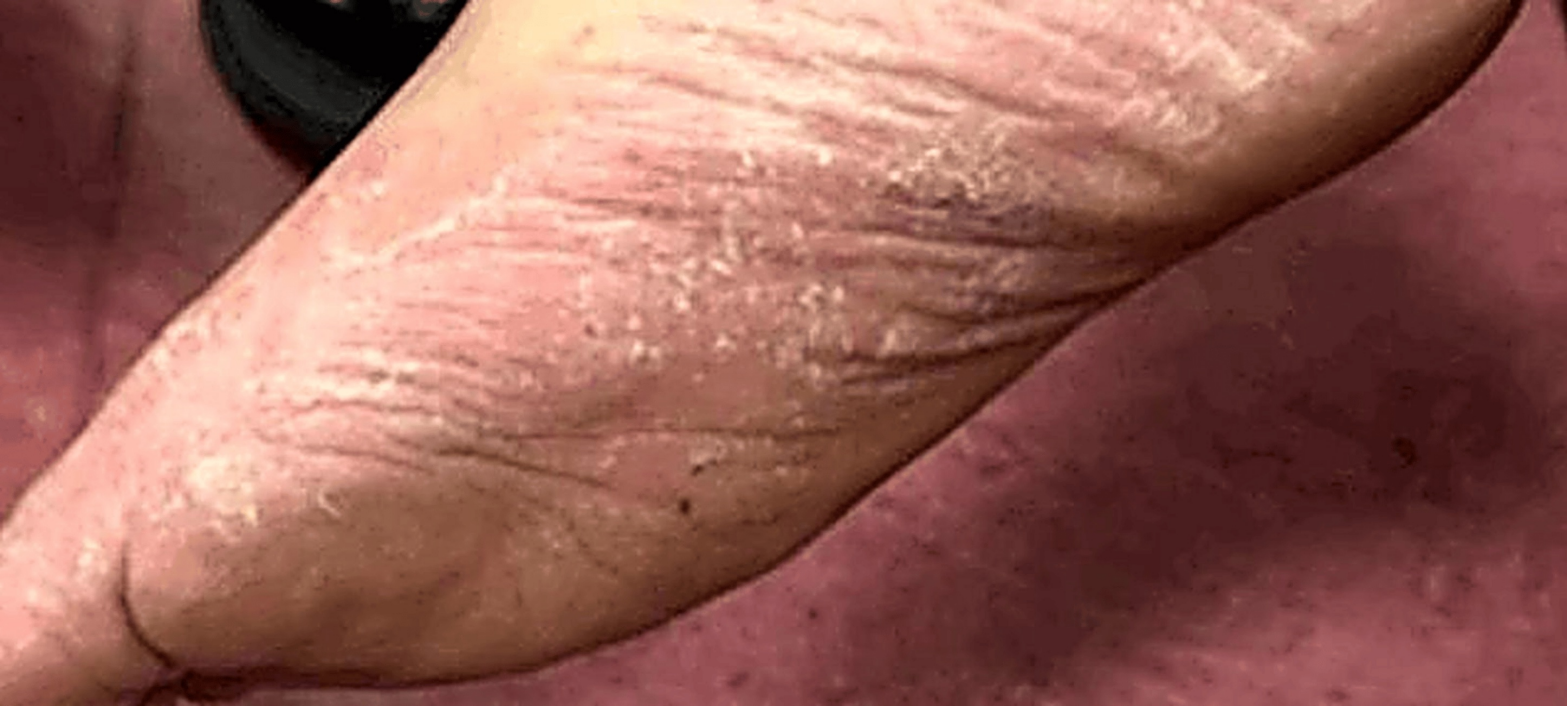
Well-defined irregular erythematous patches over the plantar surfaces of one foot, with peripheral and central scaling.

On physical examination, the patient appeared well and active. The dermatological examination revealed multiple erythematous, scaly plaques and patches scattered over the trunk, extremities (Figure 1), palms (Figure 2), and soles (Figure 3). There was no evidence of mucosal involvement, lymphadenopathy, or nail changes.

Laboratory investigations are summarized in Table 1. The vitamin D3 level is low at 17.3 nmol/L (reference range: 20-100 nmol/L), while serum IgE was within normal limits. The complete blood count and metabolic panel were largely within normal limits, except for an elevated alkaline phosphatase level at 139 U/L (reference range: 39-120) and low platelet distribution width (PDW) at 11.4% (reference range: 15.5-17.5).

**Table 1:** Laboratory investigations.

Test	Result	Reference range
Vitamin D3	17.3 nmol/L	20-100 nmol/L
Serum IgE	80.2 IU/mL	0-165 IU/mL
Alkaline phosphatase	139 U/L	39-120 U/L
Platelet distribution width (PDW)	11.4%	15.5%-17.5%

**Figure 4:**
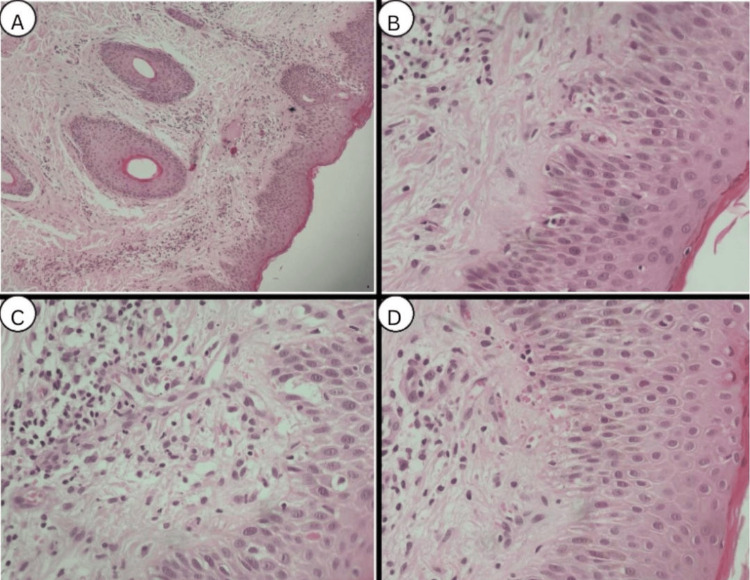
Histopathological findings from a 4 mm punch biopsy specimen stained with hematoxylin and eosin (H&E). (A) Mild epidermal acanthosis. (B) Focal spongiosis within the epidermis. (C) Superficial perivascular lymphocytic infiltrate in the papillary dermis with focal erythrocyte extravasation. (D) Focal parakeratosis in the stratum corneum.

The initial differentials included psoriasis, PR, and atopic dermatitis. KOH examination was performed and was negative for fungal elements, effectively excluding tinea infection. A 4 mm punch biopsy was taken from the left thigh for histopathological evaluation, which showed mild acanthosis, spongiosis, focal parakeratosis, and a superficial perivascular lymphocytic infiltrate with erythrocyte extravasation (Figure 4). These findings were consistent with PR in the appropriate clinical setting.

Based on the clinical presentation and histopathological findings, a diagnosis of PR was made after exclusion of other differential diagnoses. The patient was started on narrowband ultraviolet B (NBUVB) phototherapy along with topical betamethasone with calcipotriol ointment and urea 10% cream for symptomatic management. In addition, vitamin D supplementation was initiated due to documented deficiency. At follow-up three months later, the patient showed marked clinical improvement with almost complete resolution of the lesions and improvement of vitamin D levels.

## Discussion

Our patient demonstrated generalized eruption involving the trunk and extremities with prominent palmoplantar involvement and absence of a herald patch, a presentation that is not characteristic of classic PR. PR is a relatively common papulosquamous disorder that primarily affects adolescents and young adults, with a slight female predominance and possible seasonal variation. Acral or palmoplantar PR is an uncommon variant and has been reported more frequently in adults, although it may occur at any age. Only a limited number of cases have been described in pediatric patients. The morphology of acral PR varies from scaly plaques to vesicles. Atypical presentations of PR have been increasingly recognized in the literature. Management is generally similar to that of classic PR and includes symptomatic treatment with topical corticosteroids, antihistamines, and, in selected cases, phototherapy. While acral involvement may present as either primary or secondary eruptions confined to the palms and soles, lesions in other body sites such as the trunk and distal extremities may coexist [8]. This variant may create diagnostic challenges, particularly when lesions may also involve other body sites, including the trunk and extremities. The correct identification of this presentation remains essential to distinguish it from psoriasis, eczema, and secondary syphilis. Persistent PR (PPR) is defined as a disease lasting longer than 12 weeks, whereas most cases resolve spontaneously within six to eight weeks [9]. In our case, the eruption persisted for more than 12 weeks, fulfilling the definition of PPR. Such presentations require further investigation, as they may be misdiagnosed due to their uncommon features. The differential diagnosis of palmoplantar eruptions includes eczema, psoriasis, tinea, or secondary syphilis [10]. The initial differential diagnosis in our case included atopic dermatitis and psoriasis, both of which can present with scaly lesions including the acral surfaces. This necessitated performing a biopsy to confirm the diagnosis and rule out other papulosquamous disorders. Histopathological examination revealed focal parakeratosis, mild spongiosis, perivascular lymphocytic infiltrates, and extravasated erythrocytes, findings consistent with PR [11].

## Conclusions

This case highlights an uncommon presentation of PR with persistent and acral involvement in a pediatric patient. PR typically affects adolescents and young adults, with only limited reports of atypical acral involvement in pediatric patients. Recognizing atypical variants of PR is essential, as unusual distribution and prolonged disease course may lead to misdiagnosis and unnecessary investigations.

Awareness of such presentations can facilitate early diagnosis and appropriate management, thereby reducing additional diagnostic procedures. This report emphasizes the importance of maintaining PR in the differential diagnosis of palmoplantar eruptions in children, even in the absence of classic features.
